# Sums of Spike Waveform Features for Motor Decoding

**DOI:** 10.3389/fnins.2017.00406

**Published:** 2017-07-18

**Authors:** Jie Li, Zheng Li

**Affiliations:** ^1^State Key Laboratory of Cognitive Neuroscience and Learning, Beijing Normal University Beijing, China; ^2^IDG/McGovern Institute for Brain Research, Beijing Normal University Beijing, China

**Keywords:** spike sorting, decoding, neuroprosthetics, brain–machine interfaces, brain–computer interfaces

## Abstract

Traditionally, the key step before decoding motor intentions from cortical recordings is spike sorting, the process of identifying which neuron was responsible for an action potential. Recently, researchers have started investigating approaches to decoding which omit the spike sorting step, by directly using information about action potentials' waveform shapes in the decoder, though this approach is not yet widespread. Particularly, one recent approach involves computing the moments of waveform features and using these moment values as inputs to decoders. This computationally inexpensive approach was shown to be comparable in accuracy to traditional spike sorting. In this study, we use offline data recorded from two Rhesus monkeys to further validate this approach. We also modify this approach by using sums of exponentiated features of spikes, rather than moments. Our results show that using waveform feature sums facilitates significantly higher hand movement reconstruction accuracy than using waveform feature moments, though the magnitudes of differences are small. We find that using the sums of one simple feature, the spike amplitude, allows better offline decoding accuracy than traditional spike sorting by expert (correlation of 0.767, 0.785 vs. 0.744, 0.738, respectively, for two monkeys, average 16% reduction in mean-squared-error), as well as unsorted threshold crossings (0.746, 0.776; average 9% reduction in mean-squared-error). Our results suggest that the sums-of-features framework has potential as an alternative to both spike sorting and using unsorted threshold crossings, if developed further. Also, we present data comparing sorted vs. unsorted spike counts in terms of offline decoding accuracy. Traditional sorted spike counts do not include waveforms that do not match any template (“hash”), but threshold crossing counts do include this hash. On our data and in previous work, hash contributes to decoding accuracy. Thus, using the comparison between sorted spike counts and threshold crossing counts to evaluate the benefit of sorting is confounded by the presence of hash. We find that when the comparison is controlled for hash, performing sorting is better than not. These results offer a new perspective on the question of to sort or not to sort.

## Introduction

The traditional signal processing chain for cortical, penetrating-electrode brain–machine interfaces (Nicolelis, 2003; Bensmaia and Miller, 2014) consists of spike (action potential) detection, spike sorting, and decoding using sorted spike counts or spike times. The spike detection step finds time windows in the recorded voltage time series which are likely to contain action potentials. These time windows are then passed to a spike sorting algorithm, which uses the shape of the voltage vs. time curves (waveforms) in these windows to determine the identity of the neuron which emitted the spike (Wheeler and Heetderks, 1982; Lewicki, 1998; Gibson et al., 2012). The result of this classification is a neuron label for some portion, depending on recording conditions, of detected spikes, along with the time of occurrence measured in the spike detection step. Those waveforms (“hash”) which do not sufficiently match the waveforms of neurons believed to be near the electrode are traditionally discarded as they may be contaminated with noise or consist of action potentials from many neurons mixed together. The labeled spike times are then fed to the decoder for estimation of motor intention or other variables of interest. Point process decoders use spike times directly, while decoders operating on instantaneous firing rate will estimate this firing rate using spike counts in small temporal windows, computed by “binning;” alternatives to binning exist, but work similarly well (Cunningham et al., 2009).

One alternative approach to spike sorting followed by decoding is to decode using (features of) spike waveforms directly. Several studies have used this approach (Chen et al., 2012; Kloosterman et al., 2014; Todorova et al., 2014; Deng et al., 2015; Ventura and Todorova, 2015). Particularly, Ventura and Todorova (2015) proposed an innovative, computationally efficient method for incorporating waveform information in linear decoders. In this approach, features on each waveform are calculated. Then, the first p moments of these features for all spikes occurring within a time bin are calculated. This time bin can be the same size as that used for estimating instantaneous firing rate. These p moments are used, along with the threshold crossing count (a count of all detected spikes regardless of neuron label), as inputs to linear decoders such as the Kalman filter or Wiener filter. Ventura and Todorova show that this approach is comparable in accuracy to traditional spike sorting followed by decoding on binned spike counts.

In this study, we wanted to test Ventura and Todorova's approach on a larger set of offline data from two Rhesus monkeys. We also propose a modification to their approach. Instead of computing moments of features, which compute expectations, we compute the sum of the features (taken up to the p-th power). This change basically omits a division by the threshold crossing count. We show that this approach, when used with the spike amplitude, allows for better decoding accuracy than traditional sorted spike counts. We also show that using the sum instead of the moment results in significantly more accurate offline decodes, though the magnitude of differences was not large. We then analyze some design choices for the waveform feature decoding approach.

We also contribute data toward the question of whether spike sorting is beneficial (Stark and Abeles, 2007; Won, 2007; Ventura, 2008; Fraser et al., 2009; Chestek et al., 2011; Smith and Paninski, 2013; Todorova et al., 2014; Christie et al., 2015; Perel et al., 2015; Ventura and Todorova, 2015). We confirm previous findings that the hash should not be discarded, as it substantially increases decoding accuracy when included along with sorted spike counts (Todorova et al., 2014; Christie et al., 2015; Oby et al., 2016). Since sorted spike counts traditionally do not include hash, while threshold crossing counts do include hash, a comparison between these two methods is actually comparing two factors: presence of hash and use of spike sorting. We thus decided to systematically examine all four combinations of these two factors. This approach offers a new perspective on the debate within the community regarding whether spike sorting is beneficial. Previous studies comparing sorted spike counts (without hash) to threshold crossing counts were mixing the effects of two factors, so depending on the relative influences of these factors, results varied. In our data, when comparisons are made by changing only the sorting factor, the results favor spike sorting.

## Methods

### Moments of waveform features

Using features, such as amplitude or width, computed on spike waveform shapes as input for decoding is an intriguing alternative to using counts of sorted spikes. For decoders which operate on binned data, i.e., uniform time intervals in which more than one spike may occur, one challenge of applying this approach is the question of how to aggregate the features of all the spikes which were detected in a time bin, as the number of spikes in the time bin varies. The theoretical contribution by Ventura and Todorova (2015) is to show that one solution is to compute moments of the features:

(1) $$\begin{array}{l} \bar{{\text{W}}_{\text{t}}^{\text{p}}}=\frac{1}{{\text{a}}_{\text{t}}}\underset{\text{i}}{\sum }{\left(\text{f}\left({\text{s}}_{\text{i}}\right)\right)}^{\text{p}}, \end{array}$$

where $\bar{{\text{W}}_{\text{t}}^{\text{p}}}$ is the vector of sample p-th raw moments of waveform features in time bin t, a_t_ is the number of putative spikes (or threshold crossings) detected in time bin t, i indexes over these spikes, s_i_ is a single putative spike, the function f( ) computes a waveform feature vector from a spike, and the exponential on the right hand side is performed element-wise. In this section, we show that using sums is another way of aggregating waveform feature values.

We consider a single recording channel, with K neurons, at the time instant of recording a new threshold crossing. The kinematic variables (or other variables to be decoded) at the time are represented by the vector **x**. We assume each neuron's waveforms do not change with time or **x**, and so the distribution of the features computed from its waveforms is stationary. Let $\mu_{\text{j}}^{\text{p}}$ be the vector of the p-th moments of the waveform features for neuron j. For example, if we have one feature, the spike amplitude, then $\mu_{\text{j}}^{1}$ is the mean spike amplitude of neuron j. Let λ_j_(**x**) be the linear tuning function of neuron j, which gives the probability that a threshold crossing is a spike from neuron j (as opposed to other neurons or noise), given the kinematics. Let τ(**x**) be the fraction of threshold-crossings which are real spikes, as opposed to noise. Note that $\tau \left(\text{x}\right)=\underset{\text{j}}{\sum }\lambda_{\text{j}}\left(\text{x}\right)$. Let π_j_(**x**) be the probability that any one real spike is produced by neuron j, given the kinematics **x**. So π_j_ (**x**) = λ_j_(**x**) / τ(**x**), and $\underset{\text{j}}{\sum }\pi_{\text{j}}\left(\text{x}\right)=1$.

Now, let **W** be a vector random variable representing the feature values of a new threshold crossing waveform. Ventura and Todorova show that the p-th moment of **W** can be expressed as a linear function of the p-th moments of the features for each neuron and the p-th moment of the features of the noise:

(2) $$\begin{array}{l} \text{E}\left({\text{W}}^{\text{p}}|\text{x}\right)=\left[1-\tau \left(\text{x}\right)\right]{\text{w}}_{0}^{\text{p}}\;+\;\tau \left(\text{x}\right)\left[\overset{\text{K}}{\underset{\text{j}=1}{\sum }}\pi_{\text{j}}\left(\text{x}\right)\mu_{\text{j}}^{\text{p}}\right] \end{array}$$

(3) $$\begin{array}{l} ={\text{w}}_{0}^{\text{p}}\;+\;\overset{\text{K}}{\underset{\text{j}=1}{\sum }}\lambda_{\text{j}}\left(\text{x}\right)\left(\mu_{\text{j}}^{\text{p}}-{\text{w}}_{0}^{\text{p}}\right). \end{array}$$

Here, ${\text{w}}_{0}^{\text{p}}$ is the vector of the p-th moments of the features of the noise, which is assumed to be zero by Ventura and Todorova. We can satisfy this assumption by feature engineering, but we will also see in the following equation that handling a non-zero ${\text{w}}_{0}^{\text{p}}$ is done during parameter fitting. Since the λ_j_(**x**) are assumed to be linear in **x**, and $\mu_{\text{j}}^{\text{p}}$ is assumed to not change with **x**, the product $\lambda_{\text{j}}\left(\text{x}\right)\left(\mu_{\text{j}}^{\text{p}}-\;{\text{w}}_{0}^{\text{p}}\right)$ is also linear in **x**. Thus, we can write the sample moments in a time bin t as (Ventura and Todorova, Equation 2.9):

(4) $$\begin{array}{l} \bar{{\text{W}}_{\text{t}}^{\text{p}}}=\gamma_{0}^{\text{p}}\;+\;{\text{G}}^{\text{P}}{\text{x}}_{\text{t}}\;+\;\delta_{\text{t}}^{\text{p}}. \end{array}$$

Here, $\bar{{\text{W}}_{\text{t}}^{\text{p}}}$ is the sample p-th raw moment of waveform features in time bin t. **x**_t_ is the vector of kinematics at time t. $\gamma_{0}^{\text{p}}$ and **G**^P^ are a vector and a matrix of coefficients, respectively, to be fitted by linear regression. Fitting $\gamma_{0}^{\text{p}}$ and **G**^P^ takes into account any non-zero ${\text{w}}_{0}^{\text{p}}$ term. $\delta_{\text{t}}^{\text{p}}$ is the residual vector. This form is similar to the form of encoding models used in Kalman filter decoders. In those models, the left hand side is typically a sorted spike count (the observations of the Kalman filter decoder). In the waveform feature moment decoding framework, one uses waveform feature moments, along with threshold crossing counts, as observations of the Kalman filter decoder. The waveform features of different channels are included as separate observations. For example, for 100 channels and one waveform feature with three moments, the total number of waveform feature moments is 300. Combined with 100 threshold crossing counts, the total number of observations is 400.

Left-multiplying both sides of Equation (4) by the pseudoinverse of **G**^P^, (**G**^P^)^+^, we can change the equation to a form similar to the Wiener filter model, showing that we can use waveform feature moments as input to the Wiener filter as well:

(5) $$\begin{array}{l} {\gamma^{\prime }}_{0}^{\text{p}}+{\left({\text{G}}^{\text{P}}\right)}^{+}\bar{{\text{W}}_{\text{t}}^{\text{p}}}+{\delta^{\prime }}_{\text{t}}^{\text{p}}={\text{x}}_{\text{t}}. \end{array}$$

Here, ${\gamma^{\prime }}_{0}^{\text{p}}={-\left({\text{G}}^{\text{P}}\right)}^{+}\gamma_{0}^{\text{p}}$ and ${\delta^{\prime }}_{\text{t}}^{\text{p}}={-\left({\text{G}}^{\text{P}}\right)}^{+}\delta_{\text{t}}^{\text{p}}$ are new constant vectors and residual vectors, respectively. In practice, we fit the coefficients by regressing the linear model with $\bar{{\text{W}}_{\text{t}}^{\text{p}}}$ as predictor and **x**_t_ as target values, instead of using (**G**^P^)^+^ as coefficients.

### Sums of waveform features

We note that the sums of the p-th power of the features can also be expressed in a similar manner as the p-th moments of **W** (as in Equation 3):

(6) $$\begin{array}{l} \sum {\text{W}}_{\text{t}}^{\text{P}}={\text{a}}_{\text{t}}{\text{w}}_{0}^{\text{p}}+\left[\sum_{\text{j}=1}^{\text{K}}{\text{c}}_{\text{j}}\left(\text{x}\right)\left(\mu_{\text{j}}^{\text{p}}-{\text{w}}_{0}^{\text{p}}\right)\right]+\epsilon_{\text{t}}^{\text{p}}, \end{array}$$

where c_j_(**x**) is the expected spike count of neuron j in a time bin as a function of the kinematics **x**, i.e., the tuning function, and $\epsilon_{\text{t}}^{\text{p}}$ is the residual vector, which we assume to be normally distributed. We obtain Equation (6) from Equation (3) by multiplying both sides by the count of threshold crossings. Like Ventura and Todorova, we assume ${\text{w}}_{0}^{\text{p}}$ = 0. This assumption can be thought of as assuming there are no noise waveforms: every putative threshold crossing is a spike. This assumption does not lead to problems with stationary noise because any such noise can be modeled as a unit with null tuning. Assuming ${\text{c}}_{\text{j}}\left(\text{x}\right)\left(\mu_{\text{j}}^{\text{p}}\;-\;{\text{w}}_{0}^{\text{p}}\right)$ is a linear function of **x**, we see Equation (6) has a similar form as Equation (4), allowing similar use of sums of waveform features as moments of waveform features in the Kalman filter and the Wiener filter.

An advantage of using the sum approach is that the sum naturally increases with the number of spikes in a bin, and so it may be used alone, without augmenting threshold crossing counts, as input to decoders. We discuss other advantages of the sum approach in Section Moment vs. Sum in the Discussion.

### Features

In this study, we tested several simple features of waveforms for use in the waveform feature decoding framework. While it is possible to use complex features, such as the difference from a waveform template, we wanted to avoid features requiring much computation. We used the following features:

F1: Spike amplitude, i.e., voltage difference between peak and trough.
F2: Peak-to-trough-time, i.e., the time difference between the peak and trough (also called spike width).
F3: Trough, i.e., minimum voltage of the waveform.
F4: Peak, i.e., maximum voltage of the waveform.

When no spikes are recorded in a time bin, the feature values are set to zero (like Ventura and Todorova). Except for feature F4, the others are similar to the features chosen by Ventura and Todorova. We compared the different features used one at a time, and also with the first three features together, similar to Ventura and Todorova.

### Methods for comparisons

For the main analysis of overall offline decoding accuracy using waveform features, we compared the following spike data pre-processing methods:

F^*^_moment+**TC**: First three moments (no cross-moments) of features combined (by concatenation) with the threshold crossing count. The ^*^asterisk indicates which features are used, e.g., F123_moment uses the features F1, F2, and F3. This is the method proposed by Ventura and Todorova. Total count of decoder inputs per channel is M · 3 + 1, where M is the number of waveform features.
F^*^_moment: Similar to F^*^_moment+TC, but without the threshold crossing count. This pre-processing approach was not proposed by Ventura and Todorova and is not expected to do well because the moments do not directly capture the instantaneous firing rate, but we include it for completeness of comparison. Total count of inputs per channel is M · 3.
F^*^_sum: Sums of features, sums of their squares, and sums of their cubes. This is our proposed method. Total count of inputs per channel is M · 3.
F^*^_sum+**TC**: Similar to F^*^_sum, but augmented with threshold crossing counts (by concatenation). We believe the threshold crossing counts are redundant when using sums, but we include this method for completeness of comparisons. Total count of inputs per channel is M · 3 + 1.
**Sorted**: Spike counts from spike sorting by sum-of-squared-differences template matching, using experimenter created templates. Online spike sorting using these templates was performed by the Omniplex recording system. Hash are not included. Total count of inputs per channel is K, where K is the number of units decided by the experimenter.
**Threshold crossing (TC)**: The count of all threshold crossings. Total count of inputs per channel is 1.
**Sorted**+**hash**: Similar to Sorted, except that the count of putative waveforms not matching any template is included (by concatenation) as a multiunit. Total count of inputs per channel is K + 1.
**Merged**: Sorted spike counts merged together, undoing the spike sorting, but not including hash. This can be seen as threshold crossing counts without hash. Total count of inputs per channel is 1.

**Figure 1:**
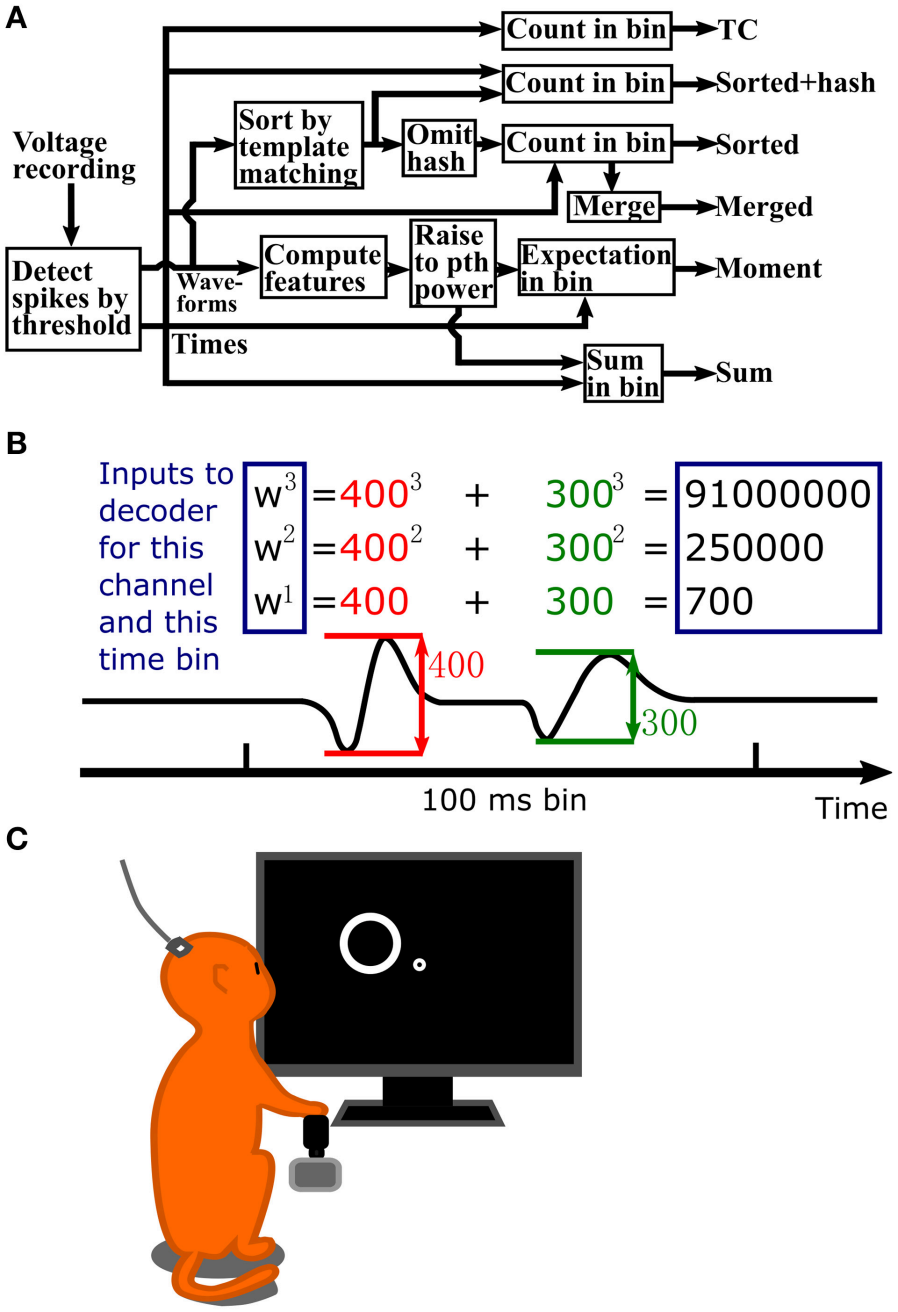
**(A)** Flowchart of signal processing. **(B)** Example waveform amplitude sum computation, up to 3rd order, for one channel and one time bin. Inputs to the decoder are in the blue rectangles. Values are normalized to unit variance before input into the decoder. **(C)** Illustration of data collection setup. A Rhesus monkey controlled a cursor with a joystick in its right hand to complete a center-out reaching task. Extracellular signals were recorded from left primary motor cortex using an implanted 96 channel Utah silicon electrode array.

A flowchart depicting the steps in signal processing for the methods we tested is shown in Figure 1A. Figure 1B illustrates the process of calculating waveform amplitude (F1) sum, up to the 3rd order, for one channel at one time bin. The full observation vector for a Kalman filter decoder at one time bin would consist of the three numbers in rectangles calculated separately for each channel. As a default, we set the number of moments or order of exponentiation (maximum value of p) to three, without using cross-moments for moment calculations. However, we also explore the effect of changing the order of exponentiation.

To compare the methods, we used them in combination with standard decoders to make offline reconstructions of previously recorded data. That is, we decode previously recorded neural activity, collected while monkeys moved their hands, into movement intentions and scored the methods by how well the decoded movements match the actual recorded hand movements. We use two standard decoders from the field. First is a position-velocity Kalman filter (Wu et al., 2006), with zero temporal offset between neural data and kinematics. This decoder used the standard linear encoding model where hand position and velocity (two dimensions each) comprise the kinematic variables. Second, we use a 3-tap Wiener filter (Carmena et al., 2003) to decode position and velocity. We used a small number of taps to reduce risk of over-fitting. Both filters used 100 ms bins, and moments or sums of features were computed with this same bin size. All input to the decoders were normalized to be zero mean and unit variance. For waveform features, this happens after the sum or moment computation. The mean and variance needed for normalization were computed on all bins of the training data.

### Animal surgeries and experiments

Neural and kinematic data were collected from two adult male Rhesus monkeys (*Macaca mulatta*) each implanted with one 96-channel Utah silicon electrode array (Blackrock Microsystems) in the left primary motor cortex over arm and hand representation areas (Li et al., 2016). All surgical procedures were in compliance with the National Institutes of Health Guide for the Care and Use of Laboratory Animals and were approved by the Institutional Animal Care and Use Committee of Beijing Normal University. Surgery was performed under sterile conditions following standard Utah array implantation procedures. Arrays were implanted ~4 mm anterior of the central sulcus, at ~15 mm left of the midline. The Utah array for monkey B (11 kg, 6 years age) had 1.0 mm long electrodes, while the Utah array for monkey M (8 kg, 4 years age) had 1.5 mm long electrodes. For more details, see Li et al. (2016).

Monkeys were trained to perform a center-out reaching task on a computer screen by using their right hands to control the computer cursor via a joystick (Figure 1C). The position of the joystick linearly mapped to the position of the cursor. The behavioral task consisted of cursor movements to center and peripheral targets (5 cm diameter) in alternation. Peripheral targets appeared at random locations equidistant from the center target location (radius varies per session, 8–10 cm). The center of the cursor had to be held within the target for a hold time (500 ms) to successfully complete the trial. Task logic, kinematic data collection, and experimental control were performed by a custom software suite (BMI3, courtesy of the Nicolelis Lab, Duke University).

Neural signals were recorded using an Omniplex (Plexon Inc.) 128-channel recording system in an electromagnetically-shielded room. Signals were amplified (up to 8,000x) and digitized (16 bit, 40 kHz) and processed online by the Omniplex system before being sent to the data acquisition computer. This processing included threshold-based spike detection (threshold set by experimenter with the goal of maximizing number of sorted units, including multiunits, mean thresholds given in Section Spike Sorting in the Results) and spike sorting using templates created by the experimenter. Both well-isolated single units and multiunits were sorted and used without distinction. The unit labels from spike sorting were used, or ignored, depending on the method, in our offline analysis. We aggressively spike sorted, preferring to differentiate groups of waveforms into more units when the choice was not obvious.

### Analysis methods

We performed all decoding and analysis offline. To make reconstructions of motor activity, we split the neural and kinematic data into training and testing portions and performed cross-validation. For Kalman filter reconstructions we used seven folds, and for Wiener filter reconstructions we used two folds, due to the computational expense of fitting the filter. We fitted parameters of the Kalman or Wiener filters using ordinary least squares (OLS) linear regression. We chose OLS because we did not want the performance trends to be affected by tuning of regularization parameters. Also, we believe plain least squares is fair because it has a built-in penalty for methods with more features (by over-fitting). An ideal sparsity-based or regularized parameter fitting procedure could decide which features are relevant, but then the optimal strategy for increasing accuracy would be to throw every imaginable feature at the decoder. This route of analysis would not give us informative results about how features and methods compare.

Accuracy of reconstructions was measured using primarily two metrics, Pearson's correlation coefficient and signal-to-noise ratio of decoding. The correlation coefficient was calculated between the reconstructed hand movement position or velocity time series (in each axis separately) and the actual hand movement position or velocity time series, as recorded by the joystick. Then, the correlations from the two axes were averaged. Signal-to-noise ratio was computed using this equation:

(7) $$\begin{array}{l} {\text{SNR}}_{\text{decoding}}=10\cdot {\text{log}}_{10}\left(\frac{\sum_{\text{t}}{\left({\text{j}}_{\text{t}}-\bar{\text{j}}\right)}^{2}}{\sum_{\text{t}}{\left({\text{j}}_{\text{t}}-{\text{r}}_{\text{t}}\right)}^{2}}\right), \end{array}$$

where j_t_ is the joystick position or velocity in one axis at time t, $\bar{\text{j}}$ is the mean value, r_t_ is the reconstructed value at time t, and SNR_decoding_ is in decibels (dB). The SNR of the two axes were then averaged. We include the SNR alongside the traditional correlation coefficient since it measures errors in scale and offset and does not saturate. It can be thought of as the normalized inverse of the mean-squared-error with a log transform. We also used mean-squared-error in one analysis so as to have the same metric as Ventura and Todorova. We average across cross-validation folds, for one accuracy value per recording session.

For quantifying the quality of cortical recordings, we computed the signal-to-noise ratio of recording, which measures the size of spikes compared to the size of the background noise:

(8) $$\begin{array}{l} {\text{SNR}}_{\text{recording}}\;=20\cdot {\text{log}}_{10}\left(\frac{\text{P}}{\text{SD}}\right), \end{array}$$

where P is the mean peak-to-trough height of spike waveforms sorted on the channel, SD is the standard deviation of the noise on the channel (when no spikes are present), and SNR_recording_ is in decibels (dB). We calculate SNR of recording as averages across channels for each session.

We tested for significant differences using paired, two-tailed sign tests with significance level α = 0.05. In most of the analysis, the pairs of methods compared were pre-planned, but in exploratory analysis we used correction for multiple comparisons: the Holm-Bonferroni method. We computed up to 16 numbers or measurements for each spike processing method (2 monkeys × 2 filters {Kalman, Wiener} × 2 variables {position, velocity} × 2 metrics {CC, SNR_decoding_}), which can be viewed as 16 experiments. All of these measurements are tabulated for completeness. For some analyses we only computed a subset of these measurements for the sake of speed. The trends in the measurements were generally consistent, with no interesting deviations. In the Results Section and figures, we show the correlation coefficient of Kalman filter reconstructions of velocity. We do this to reduce clutter and focus the analyses. These numbers were representative of the trends in all the measurements, and the position-velocity Kalman filter, velocity control, and evaluation by correlation coefficient are commonly-used in this field.

We analyzed data from 47 recording sessions from monkey B and 28 recording sessions from monkey M, chosen from all available data by the criteria of: (1) free from large recording noise or artifacts, (2) dates of recording have roughly equal-length time gaps in between, with the intention of sampling a range of recording quality. Data used from sessions ranged from 5.46 to 8.33 min in length (mean 8.24). In terms of trials of the center-out task, the data analyzed were 131–199 trials in length (mean 197). Data from monkey B were collected 10–184 days post-implantation, and data from monkey M were collected 15–168 days post-implantation.

## Results

We computed the offline reconstruction accuracies for two monkeys, for the Kalman and Wiener filters, for position and velocity, and using correlation coefficient and signal-to-noise ratio as metrics, for a total of 16 mean values per method. The results are tabulated in Table 1 as mean ± SEM. In the results below we focus on the Kalman filter velocity reconstruction accuracy as measured by correlation, as this is a commonly-used decoding approach. The trends in the other measurements were similar.

**Table 1:** Mean ± SEM of decoding accuracy.

	**Sorted**	**Sorted +hash**	**Merged**	**TC**	**F123_moment**	**F123_moment + TC**	**F123_sum**	**F123_sum + TC**	**F1_sum**	**F1_sum +TC**
BPKC	0.820 ± 0.011	0.852 ± 0.008	0.796 ± 0.012	0.823 ± 0.010	0.768 ± 0.015	0.822 ± 0.012	0.826 ± 0.012	0.841 ± 0.009	0.853 ± 0.009	0.846 ± 0.010
BPKS	4.816 ± 0.203	5.547 ± 0.201	4.299 ± 0.204	4.808 ± 0.208	3.895 ± 0.245	4.958 ± 0.248	5.008 ± 0.278	5.261 ± 0.240	5.599 ± 0.217	5.412 ± 0.288
BVKC	0.744 ± 0.010	0.768 ± 0.009	0.724 ± 0.012	0.746 ± 0.011	0.659 ± 0.015	0.718 ± 0.012	0.729 ± 0.013	0.754 ± 0.009	0.767 ± 0.009	0.760 ± 0.011
BVKS	3.576 ± 0.152	3.906 ± 0.147	3.297 ± 0.163	3.554 ± 0.164	2.446 ± 0.190	3.160 ± 0.190	3.244 ± 0.224	3.590 ± 0.190	3.894 ± 0.159	3.707 ± 0.224
BPWC	0.687 ± 0.016	0.744 ± 0.013	0.648 ± 0.016	0.694 ± 0.014	0.646 ± 0.015	0.735 ± 0.014	0.737 ± 0.014	0.740 ± 0.013	0.741 ± 0.013	0.744 ± 0.013
BPWS	2.858 ± 0.168	3.587 ± 0.182	2.417 ± 0.152	2.902 ± 0.162	2.372 ± 0.136	3.437 ± 0.179	3.455 ± 0.181	3.506 ± 0.181	3.507 ± 0.182	3.541 ± 0.181
BVWC	0.637 ± 0.013	0.673 ± 0.011	0.615 ± 0.013	0.642 ± 0.012	0.566 ± 0.014	0.659 ± 0.012	0.670 ± 0.011	0.672 ± 0.011	0.673 ± 0.011	0.675 ± 0.011
BVWS	2.369 ± 0.114	2.711 ± 0.114	2.178 ± 0.109	2.408 ± 0.112	1.764 ± 0.101	2.578 ± 0.121	2.692 ± 0.118	2.715 ± 0.118	2.725 ± 0.120	2.747 ± 0.119
MPKC	0.844 ± 0.008	0.879 ± 0.008	0.831 ± 0.010	0.872 ± 0.007	0.804 ± 0.014	0.851 ± 0.012	0.870 ± 0.007	0.882 ± 0.006	0.883 ± 0.006	0.881 ± 0.006
MPKS	5.176 ± 0.215	6.291 ± 0.228	4.871 ± 0.229	5.918 ± 0.235	4.324 ± 0.307	5.527 ± 0.316	5.999 ± 0.221	6.314 ± 0.208	6.328 ± 0.209	6.252 ± 0.218
MVKC	0.738 ± 0.008	0.783 ± 0.007	0.726 ± 0.009	0.776 ± 0.007	0.682 ± 0.013	0.740 ± 0.013	0.764 ± 0.009	0.783 ± 0.006	0.785 ± 0.006	0.783 ± 0.006
MVKS	3.375 ± 0.109	4.094 ± 0.115	3.203 ± 0.118	3.925 ± 0.108	2.491 ± 0.257	3.269 ± 0.270	3.729 ± 0.172	4.099 ± 0.105	4.104 ± 0.104	4.061 ± 0.104
MPWC	0.731 ± 0.013	0.795 ± 0.012	0.714 ± 0.014	0.783 ± 0.012	0.724 ± 0.014	0.799 ± 0.012	0.804 ± 0.012	0.804 ± 0.012	0.804 ± 0.012	0.806 ± 0.011
MPWS	3.251 ± 0.201	4.271 ± 0.248	3.022 ± 0.214	4.056 ± 0.243	3.173 ± 0.200	4.374 ± 0.254	4.447 ± 0.251	4.454 ± 0.251	4.444 ± 0.253	4.470 ± 0.252
MVWC	0.623 ± 0.012	0.692 ± 0.010	0.609 ± 0.013	0.683 ± 0.011	0.608 ± 0.010	0.693 ± 0.010	0.702 ± 0.010	0.702 ± 0.010	0.703 ± 0.011	0.704 ± 0.010
MVWS	2.144 ± 0.112	2.850 ± 0.124	2.021 ± 0.120	2.776 ± 0.127	2.025 ± 0.087	2.874 ± 0.124	2.991 ± 0.127	2.997 ± 0.127	3.013 ± 0.130	3.019 ± 0.129
CC average	0.728	0.773	0.708	0.752	0.682	0.752	0.763	0.772	0.776	0.775
CC Rank	8	3	9	6	10	7	5	4	1	2
SNR average	3.45	4.16	2.92	3.79	2.81	3.77	3.95	4.12	4.20	4.15

*Each column is a method, see Section Methods for Comparisons for details. Each row is an accuracy measure, with the following name code: first letter, monkey {B,M}; second letter, kinematic variable {position, velocity}; third letter, filter {Kalman, Wiener}; fourth letter, metric {correlation, signal-to-noise ratio of decoding, dB}. CC average, average of all correlation measures; SNR average, average of all SNR measures*.

### Sum of waveform amplitude

In this study, we wanted to gather more data about the potential efficacy of using waveform features for motor decoding as an alternative paradigm for brain-machine interfaces. We also wanted to compare our proposed modification of using sums of features instead of moments. We were expecting waveform feature decoding to not significantly differ from decoding by sorted spike counts, consistent with the results from Ventura and Todorova (2015).

**Figure 2:**
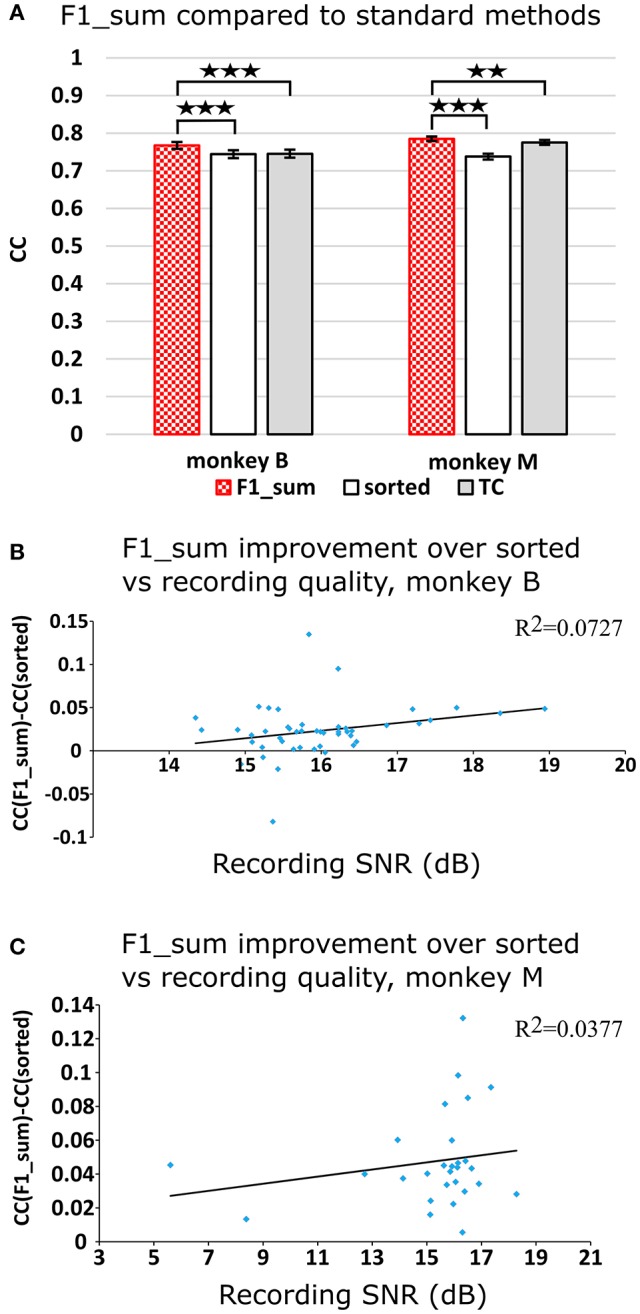
**(A)** Offline reconstruction accuracy comparison of amplitude sum (F1_sum) with traditional methods. Correlations of velocity reconstructions via Kalman filter are plotted. Error bars indicate ± SEM. **(B)** Differences in accuracy between F1_sum and sorted spike counts regressed over signal-to-noise ratio of recordings, showing relative sensitivity of F1_sum performance to recording quality. Each data point is a session. Line is best fit linear trend. Monkey B shown. **(C)** Same for monkey M. ^**^p < 0.001; ^***^p < 0.0001.

Figure 2A plots the Kalman filter velocity reconstruction accuracy as measured by correlation when using sorted spike counts (sorted), threshold crossings (TC), and sum of the waveform amplitude feature (F1_sum) as pre-processing. In this measure, F1_sum had mean *r*-values of 0.767, 0.785 (monkey B, M), sorted had mean *r*-values of 0.744, 0.738, and TC had mean *r-*values of 0.746, 0.776. The mean correlation (across all measures using correlation) and mean SNR (across all measures using SNR) are given at the bottom of Table 1. We were surprised to find that using the waveform amplitude sums for decoding actually resulted in significantly higher correlation than that when using sorted spike counts or threshold crossings (*P*-values in Supplementary Table A), even though the magnitudes of differences were small.

We were curious whether waveform feature decoding was less robust to recording quality degradation as compared to decoding from sorted units. We regressed (F1_sum − sorted), as the dependent variable vs. the quality of recording (signal-to-noise ratio of recording), as the independent variable. The correlations were nominally positive but not significant (monkey B: *p* = 0.0669, slope = 0.0088, *R*^2^ = 0.0727; monkey M: *p* = 0.322, slope = 0.0021, *R*^2^ = 0.0377; Figures 2B,C). This suggests that amplitude sum was not more sensitive to recording quality than template-based spike sorting.

### Comparison to previous work

**Figure 3:**
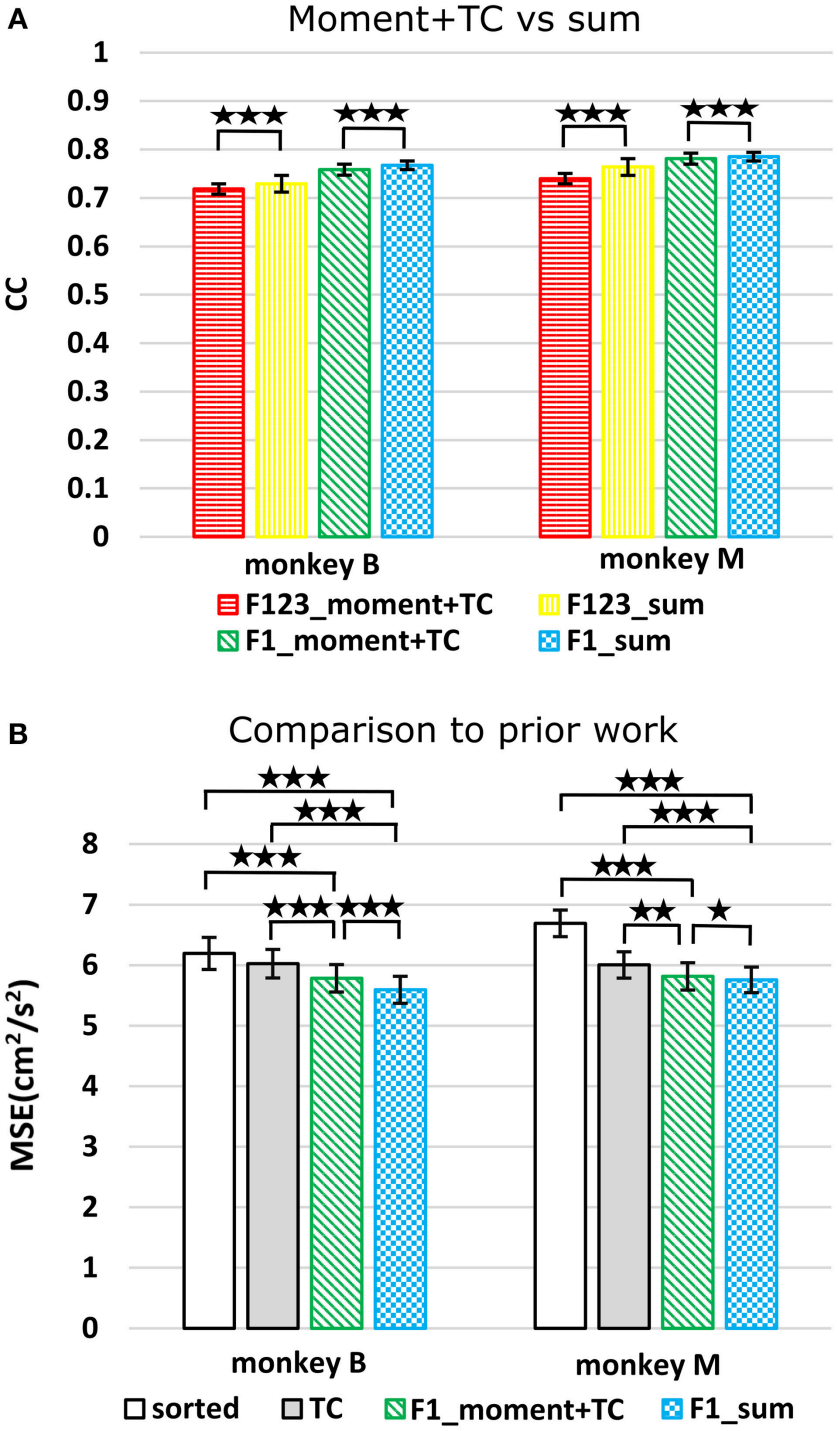
**(A)** Offline reconstruction accuracy comparison between moment and sum. Correlations of velocity reconstructions via Kalman filter are plotted. Error bars indicate ±SEM. **(B)** For comparison to Ventura and Todorova, accuracy was also measured in mean-squared-error. Smaller values are better. ^*^p < 0.05; ^**^p < 0.001; ^***^p < 0.0001.

We compared using moments of waveform features (augmented by threshold crossing counts) vs. using sums of waveform features (Figure 3A, *p*-values in Supplementary Tables B,C). Decoding using F123_sum resulted in significantly higher correlation compared to decoding using F123_moment+TC. Decoding using F1_sum (amplitude sum) resulted in significantly higher correlation compared to decoding using F1_moment+TC. Though significant, the magnitudes of differences were small.

We performed further analysis with the amplitude feature alone. To use the same metrics as Ventura and Todorova (2015), we computed the mean-squared-error (MSE) on the Kalman filter decoded velocity. Our F1_moment+TC condition is the same as their model 1. Reconstruction accuracies in MSE, efficiency relative to TC, i.e., MSE(TC)/MSE(*), and CC are tabulated in Table 2, and MSE-values are plotted in Figure 3B. Significance testing (*p*-values in Supplementary Table C) indicated that F1_moment+TC was significantly better than sorted and threshold crossing for both the correlation and MSE measures (consistent with their findings). Testing also showed F1_sum was significantly better than sorted and threshold crossing for both the correlation and MSE measures. F1_sum was significantly better than F1_moment+TC in the MSE measure.

**Table 2:** Comparison with previous work.

	**Sorted**	**Sorted +hash**	**TC**	**F1_moment**	**F1_moment + TC**	**F1_sum**	**F1_sum+TC**
BVKM	6.193 ± 0.265 (0.973)	5.665 ± 0.213 (1.063)	6.023 ± 0.236 (1)	7.061 ± 0.266 (0.853)	5.782 ± 0.226 (1.042)	5.592 ± 0.223 (1.077)	6.000 ± 0.417 (1.004)
MVKM	6.689 ± 0.220 (0.897)	5.773 ± 0.201 (1.040)	6.002 ± 0.217 (1)	6.906 ± 0.230 (0.869)	5.814 ± 0.225 (1.032)	5.756 ± 0.212 (1.043)	5.764 ± 0.207 (1.041)
BVKC	0.744 ± 0.010	0.768 ± 0.009	0.746 ± 0.011	0.695 ± 0.012	0.758 ± 0.009	0.767 ± 0.009	0.760 ± 0.011
MVKC	0.738 ± 0.008	0.783 ± 0.007	0.776 ± 0.007	0.719 ± 0.006	0.781 ± 0.006	0.785 ± 0.006	0.783 ± 0.006

Entries show mean ± SEM of decoding accuracy. Numbers in parentheses indicate efficiency relative to TC, i.e., MSE(TC)/MSE(^*^). Each column is a method. Each row is an accuracy measure, with the following name code: first letter, monkey {B,M}; second letter, kinematic variable {position, velocity}; third letter, filter {Kalman}; last letter, metric {correlation, mean-squared-error}. MSE units are (cm/s)^2^, with smaller values better. Larger relative efficiencies are better.

### Feature comparison

**Figure 4:**
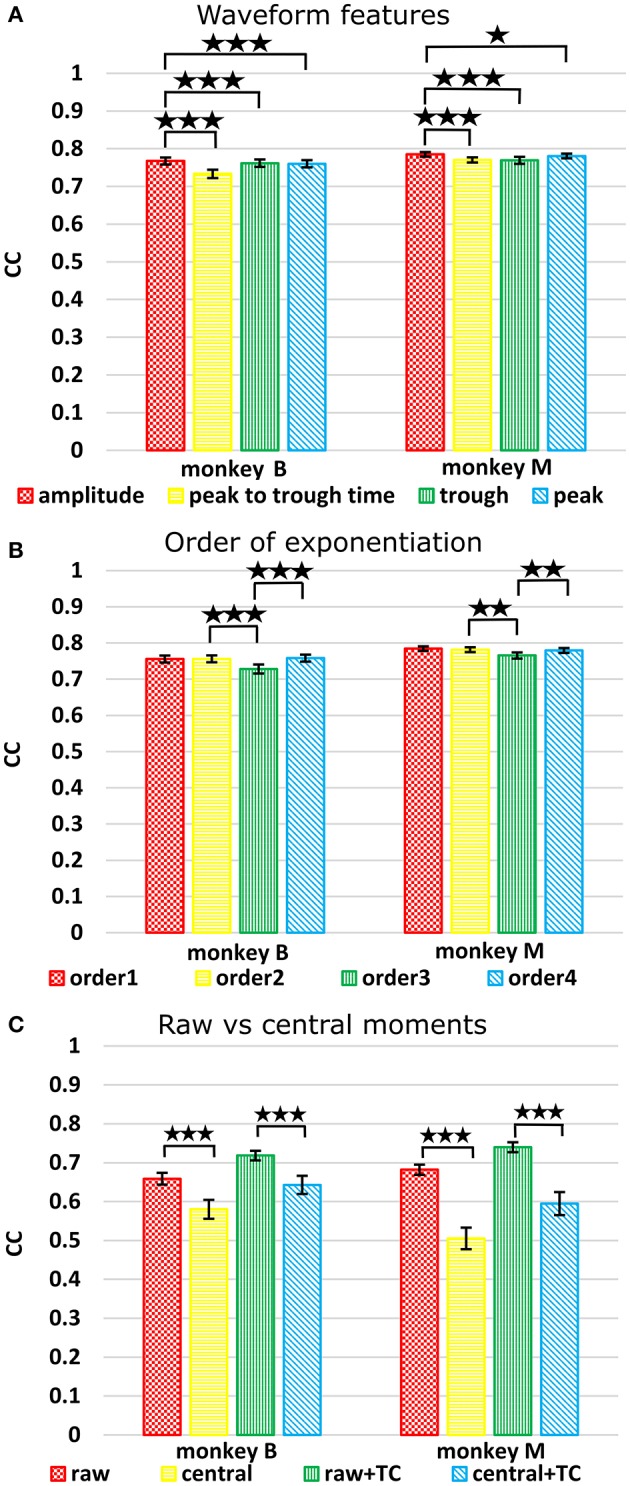
**(A)** Comparison between waveform features under sum approach. Correlations of velocity reconstructions via Kalman filter are plotted. Error bars indicate ±SEM. **(B)** Comparison between orders (degree to exponentiate feature values) of sums. **(C)** Comparison between raw and central moments. ^*^p < 0.05; ^**^p < 0.001; ^***^p < 0.0001.

We next compared the four waveform features under the sum paradigm. Each feature and its powers up to the 3rd order were summed and used without threshold crossing counts. Table 3 and Figure 4A show the resulting reconstruction accuracy. For this analysis, we compared only Kalman filter position and velocity reconstruction accuracy, as measured by correlation coefficient, and plot only the velocity results.

**Table 3:** Mean ± SEM of decoding accuracy when using different features.

	**Amplitude**	**Peak-to-trough-time**	**Trough**	**Peak**
BPKC	0.853 ± 0.009	0.813 ± 0.011	0.844 ± 0.010	0.846 ± 0.009
BVKC	0.767 ± 0.009	0.733 ± 0.011	0.762 ± 0.010	0.760 ± 0.010
MPKC	0.883 ± 0.006	0.869 ± 0.007	0.864 ± 0.010	0.878 ± 0.006
MVKC	0.785 ± 0.006	0.770 ± 0.007	0.769 ± 0.009	0.781 ± 0.006

*Each column is a method, each row is an accuracy measure, with the following name code: first letter, monkey {B,M}; second letter, kinematic variable {position, velocity}; third letter, filter {Kalman}; fourth letter, metric {correlation}*.

The features ranked by average correlation (average of position and velocity), from best to worst, were: (1) amplitude (F1_sum), (2) peak voltage (F4_sum), (3) trough voltage (F3_sum), and (4) peak-to-trough-time (F2_sum). Significance testing results and *p*-values corrected using the Holm-Bonferroni method are given in Supplementary Table D. Amplitude (F1) was significantly better than peak-to-trough-time (F2), trough (F3), and peak (F4), but generally differences were small.

### Order of exponentiation

We next compared performance between different orders (p) of the waveform features. We raised features to the first, second, third, or fourth power before summation, analogous to using the first one, two, three, or four moments. Note that the 2nd order condition includes the features raised to the 1st power; the 3rd order condition includes the features raised to the 1st and 2nd power, and so on. For this analysis we used features F1 (amplitude), F2 (spike width), and F3 (trough). Table 4 shows the resulting Kalman filter position and velocity reconstruction accuracy measured by correlation coefficient, and Figure 4B plots the velocity accuracy.

**Table 4:** Mean ± SEM of decoding accuracy when exponentiating features F1, F2, and F3 to different orders and summing.

	**Order 1**	**Order 2**	**Order 3**	**Order 4**
BPKC	0.838 ± 0.009	0.845 ± 0.009	0.824 ± 0.012	0.846 ± 0.009
BVKC	0.756 ± 0.010	0.756 ± 0.009	0.728 ± 0.012	0.758 ± 0.010
MPKC	0.881 ± 0.006	0.882 ± 0.006	0.871 ± 0.007	0.879 ± 0.006
MVKC	0.785 ± 0.006	0.782 ± 0.007	0.765 ± 0.009	0.779 ± 0.007

*Each column is a method, each row is an accuracy measure, with the following name code: first letter, monkey {B,M}; second letter, kinematic variable {position, velocity}; third letter, filter {Kalman}; fourth letter, metric {correlation}*.

The accuracy was very similar amongst the orders. The ranking of average correlation (average of position and velocity) from best to worst was: (1) 2nd order, (2) 4th order, (3) 1st order, and (4) 3rd order. Holm-Bonferroni-corrected significance testing results and *p*-values are tabulated in Supplementary Table E.

### Raw vs. central moments

Ventura and Todorova used raw moments in their design (the above analyses all used raw moments). We were curious if using central moments, instead of raw moments, could improve the performance of waveform feature moments. To be specific, when computing raw moments, one does not first subtract the mean value:

(9) $$\begin{array}{l} {\text{raw}}_{\text{p}}\;=\text{E}\left[{\text{W}}^{\text{p}}\right], \end{array}$$

where p is the order of the moment. When computing central moments (of order 2 and above), one first subtracts the mean value:

(10) $$\begin{array}{l} {\text{central}}_{\text{p}}\;=\text{E}\left[{\left(\text{W}-\text{E}\left[\text{W}\right]\right)}^{\text{p}}\right]. \end{array}$$

To avoid giving the decoder garbage values, for the 1st order central moment, we used the 1st order raw moment. We compared raw and central moments of features F1 (amplitude), F2 (spike width), and F3 (trough) of order 3, without cross-moments, and both with and without including threshold crossing counts. Kalman filter position and velocity reconstruction accuracies, as measured by correlation, are tabulated in Table 5, and velocity reconstruction correlation values are plotted in Figure 4C.

**Table 5:** Mean ± SEM of decoding accuracy, comparing raw vs. central moments.

	**Raw**	**Raw+TC**	**Central**	**Central+TC**
BPKC	0.768 ± 0.015	0.822 ± 0.012	0.660 ± 0.027	0.738 ± 0.025
BVKC	0.659 ± 0.015	0.718 ± 0.012	0.580 ± 0.024	0.643 ± 0.023
MPKC	0.804 ± 0.014	0.851 ± 0.012	0.615 ± 0.032	0.699 ± 0.033
MVKC	0.682 ± 0.013	0.740 ± 0.013	0.505 ± 0.028	0.595 ± 0.030

*Each column is a method, each row is an accuracy measure, with the following name code: first letter, monkey {B,M}; second letter, kinematic variable {position, velocity}; third letter, filter {Kalman}; fourth letter, metric {correlation}*.

Significance testing (*p*-values in Supplementary Table F) indicated that raw moments without TC were significantly better than central moments without TC, and raw moments with TC were also significantly better than central moments with TC.

### Spike sorting

We compared decoding using sorted spike counts vs. unsorted counts, a long-running question in the field. Spike sorting typically rejects threshold crossings which do not match any unit's template or criteria. However, since these hash waveforms may be multiunit waveforms or single-unit waveforms contaminated with noise, they may potentially help decoding. Todorova et al. (2014) found that including (concatenating) the count of this hash improved the decoding accuracy of sorted spike counts. We note that when comparing sorted spike counts without hash vs. threshold crossings, one is not just comparing the utility of spike sorting, since the hash is included in threshold crossings but not in the sorted spike counts. Thus, to properly evaluate the benefit of spike sorting, we compare sorted spike counts with hash vs. threshold crossings. We also compare sorted spike counts (without hash) with a “merged” condition, derived by merging the units separated by spike sorting, which is equivalent to threshold crossings which do not include the hash.

**Figure 5:**
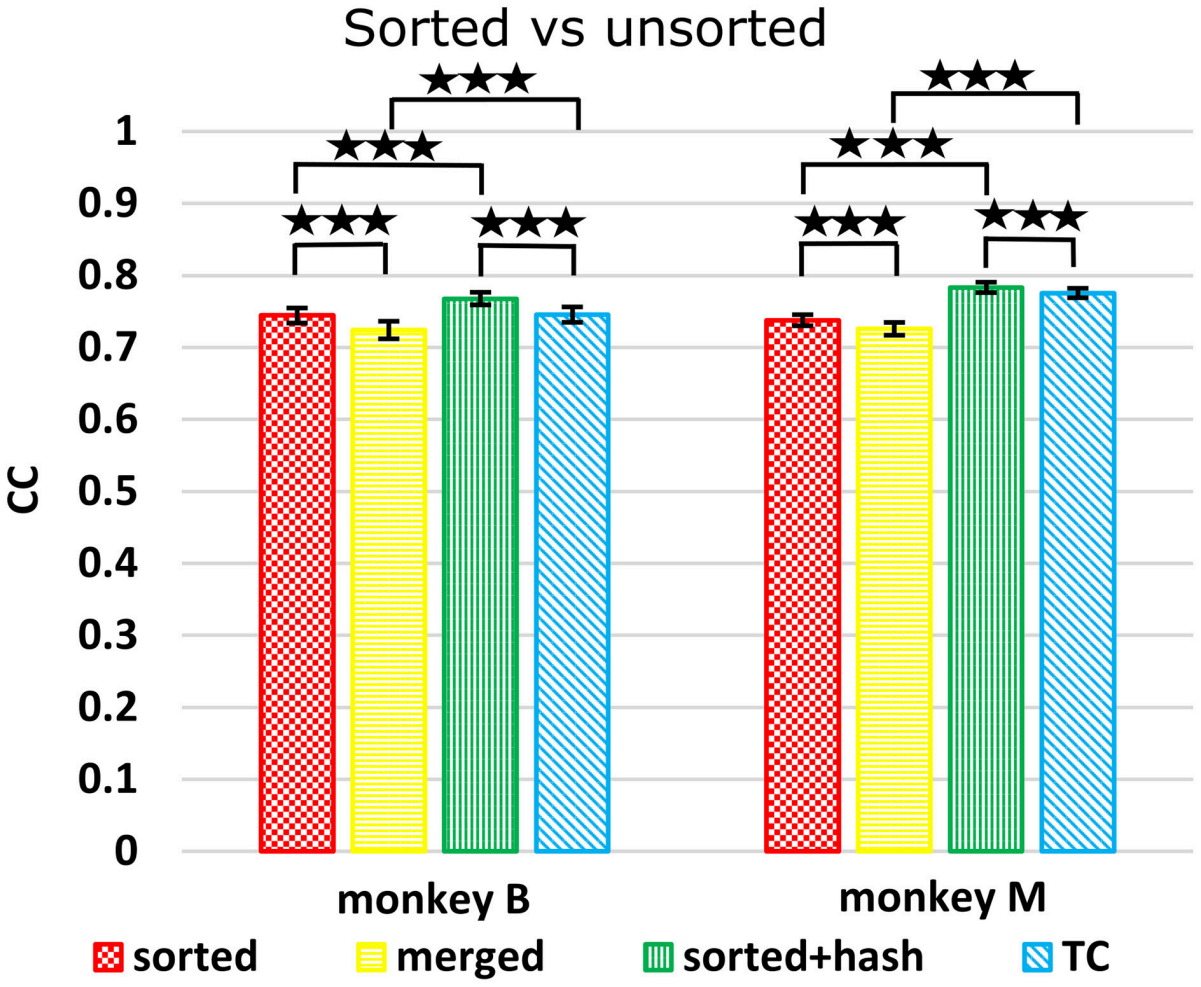
Comparison between sorted and unsorted. Correlations of velocity reconstructions via Kalman filter are plotted. Error bars indicate ±SEM. Hash are waveforms which do not match any units' template. Merged condition is sorted spike counts with units merged back together, equivalent to threshold crossing counts without hash. ^***^p < 0.0001.

Figure 5 shows Kalman filter velocity reconstruction results as measured by correlation while Table 1 shows all measures. Significance testing (*p*-values in Supplementary Table G) indicated that sorted+hash was in all 16 measures significantly better than threshold crossing. Furthermore, sorted was in all 16 measures significantly better than merged.

In the (confounded, in our opinion) comparison between sorted and threshold crossing, 8 of 16 comparisons were significantly different (in favor of threshold crossing) while others were not significantly different. The comparisons which were significant were all from monkey M, and none of the comparisons for monkey B were significant. The two monkeys' electrode lengths were different and different experimenters collected the data, set thresholds, and made templates for the spike sorting. The mean (across sessions and channels) spike detection thresholds in our data were −2.84 ± 0.22 and −2.77 ± 0.50 times the noise standard deviation for monkey B and monkey M, respectively. The mean (across sessions and channels) signal-to-noise ratios of our recordings were 16.0 ± 0.92 and 15.2 ± 2.6 dB for monkey B and monkey M, respectively. The ± indicate standard deviation across sessions.

Though not a main point of our study, we found that sorted+hash was in all measures significantly better than sorted, replicating Todorova et al. (2014). Threshold crossing was in all measures significantly better than merged. These two results show that the useful information in the hash was more beneficial than the detrimental effects of the noise in the hash in our recordings and for our threshold values.

### Analysis under more stringent spike detection threshold

We wanted to know to what extent our results generalized to a more stringent spike detection threshold, as our average spike detection thresholds were less stringent than commonly seen in the field. Thus, we repeated a subset of the analyses using a threshold of −4.5 times the noise standard deviation for all channels. We applied the new threshold by culling spikes which did not meet the threshold. The spike sorting labels, if applicable, were not altered. We performed reconstructions with the Kalman filter and measured accuracy in terms of correlation of velocity estimates with actual velocities.

Correlation of reconstructions are tabulated in Table 6. Compared to reconstructions under our original thresholds, the reconstructions under −4.5 thresholds were all significantly and substantially less accurate (*p*-values in Supplementary Table H). Under −4.5 thresholds, F1_sum was still significantly better than sorted, but no longer better than sorted+hash and TC. However, comparing F1_sum under original thresholds vs. sorted, sorted+hash, and TC under −4.5 thresholds shows that F1_sum was significantly better.

**Table 6:** Mean ± SEM of decoding accuracy when using −4.5 noise standard deviations as spike detection threshold, with accuracy at original thresholds also given for comparison.

	**Sorted**	**Sorted +hash**	**Merged**	**TC**	**F1_sum**	**F2_sum**	**F3_sum**	**F4_sum**	**F1_moment +TC**
BVKC (−4.5)	0.671 ± 0.015	0.745 ± 0.010	0.670 ± 0.014	0.710 ± 0.013	0.685 ± 0.021	0.653 ± 0.023	0.676 ± 0.020	0.673 ± 0.021	0.630 ± 0.024
BVKC (avg. −2.84)	0.744 ± 0.010	0.768 ± 0.009	0.724 ± 0.012	0.746 ± 0.011	0.767 ± 0.009	0.733 ± 0.011	0.762 ± 0.010	0.760 ± 0.010	0.758 ± 0.009
MVKC (−4.5)	0.566 ± 0.023	0.756 ± 0.007	0.564 ± 0.026	0.703 ± 0.017	0.630 ± 0.029	0.565 ± 0.036	0.610 ± 0.032	0.593 ± 0.034	0.455 ± 0.046
MVKC (avg. −2.77)	0.738 ± 0.008	0.783 ± 0.007	0.726 ± 0.009	0.776 ± 0.007	0.785 ± 0.006	0.770 ± 0.007	0.769 ± 0.009	0.781 ± 0.006	0.781 ± 0.006

*Each column is a method, each row is an accuracy measure, with the following name code: first letter, monkey {B,M}; second letter, kinematic variable {velocity}; third letter, filter {Kalman}; fourth letter, metric {correlation}; parentheses, spike detection threshold in noise standard deviations*.

Under −4.5 thresholds, F1_sum was still significantly better than F1_moment+TC. Amplitude was still the best feature nominally in terms of correlation, though some testing results were no longer significant.

In terms of sorted vs. unsorted comparisons, under −4.5 thresholds, sorted+hash was still significantly better than TC, though sorted was not significantly different from merged. The benefit of including hash was still significant.

## Discussion

In this study, we contributed more experimental data toward validating the moments of waveform features decoding approach. We proposed a modification of the moments approach which uses sums, and show it facilitates small, but significant improvements in offline decoding accuracy. We show that using the sums of waveform amplitudes to decode offline allows accuracy which is significantly better than both sorted spike counts and threshold crossing counts. We compare different choices of waveform features, different values for the order of exponentiation, and raw vs. central moments. Lastly, we present data comparing the use of sorted vs. unsorted spike counts, in which the presence of hash waveforms is controlled, finding that sorted spike counts are significantly better for decoding.

### Waveform amplitude sums

Our results showed that using the sums of waveform amplitude facilitated decoding that was significantly more accurate than sorted spike counts or threshold crossing counts, the two commonly-used approaches for motor decoding. Had our results shown no significant difference between this new paradigm and the traditional methods, the new approach would already be a viable alternative to the traditional methods (for reasons we discuss below). Our results with two decoders, two output variables, two accuracy metrics, and two monkeys show a consistent and significant improvement. Even though the absolute improvements were small in terms of correlation, in terms of signal-to-noise ratio of decoding accuracy, an average improvement of 0.75 dB vs. sorted (see last row of Table 1) and 0.41 dB vs. threshold crossings correspond to ~16% and 9% reduction in mean-squared-error, respectively, which are substantial improvements. Even though threshold crossings and sorted spikes with hash were better than sums of waveform amplitude when spike detection was performed with −4.5 noise standard deviation thresholds, sums of waveform amplitude at our original spike detection thresholds was still better than both of these methods at −4.5, so in terms of accuracy, there is little reason to use the traditional methods with −4.5 thresholds.

The sums of waveform features paradigm offers some advantages over the spike sorting approach: (1) no templates or sorting parameters need to be set, an operation which requires substantial experimenter time or advanced algorithms. (2) Depending on the details of the sorting algorithm, waveform features may be less computationally expensive. For example, compared to template matching on sum-of-squared differences, which is 3·*s*·*K*+*K* floating point operations per waveform, where *s* is the number of samples per waveform and *K* is the number of units, amplitude sum is 2·*s* operations per waveform. (3) Instead of updating sorting parameters to follow changes in waveform shape or after detecting loss or emergence of neurons, parameters of the decoder can be updated to capture these changes, using previously published adaptive methods (e.g., Li et al., 2011). This approach would combine updates for spike sorting parameters and decoding parameters into one operation.

One disadvantage for waveform feature decoding is that emergence of new neurons or loss of neurons cannot be detected during spike sorting and used as triggers for parameter updates. This may not matter if decoder parameter updates occur in the background at all times regardless of spike sorting instability. Alternatively, a separate monitoring process using spike sorting can be used to detect such changes. Since such a monitor would not have to process spikes in real time for decoding, it can operate intermittently in the background, consuming few computational resources.

Compared to threshold crossings, the sums of waveform features paradigm is not faster in execution. However, our work is fairly preliminary in terms of developing this approach, so there is much room for improvement, while the threshold crossing approach is unlikely to be improved any further without also increasing complexity. From a theoretical view, using only the threshold crossing count is statistically inefficient, as waveform shape information is ignored (Ventura and Todorova, 2015).

### Moment vs. sum

The comparisons between moments+TC and sums show that sums were significantly better, though the magnitude of differences were small. The comparison remained the same when more stringent spike detection thresholds were used. The small sizes of the differences mean that practically, the difference between approaches was minor on our data. However, we believe the sum approach has some theoretical advantages.

First, using sums do not require the inclusion of threshold crossing counts. This allows lower dimensional input to decoders and thus reduces the risk of over-fitting. Second, since threshold crossing counts are not required, this approach has the potential to be extended: sums of features computed directly from voltage traces, without detection of spikes. This is a direction of our future work. Third, we believe the definition of tuning curves under the sums framework is slightly more intuitive. Under the sums framework, tuning functions output the expected number of spikes in the time bin, which is the classic rate coding approach. Under the moments framework, tuning functions output the probability that a detected threshold crossing is from the neuron in question, i.e., a relative activation. The slight awkwardness with this definition is that this probability depends on the activity of the other neurons and the amount of noise on the channel, as probabilities have to sum to 1.

### Spike sorting

We contribute data on a question of interest to the brain-machine interface community: whether spike sorting is beneficial. This is often framed as a choice between whether sorted spike counts are better (Won, 2007; Smith and Paninski, 2013; Todorova et al., 2014; Perel et al., 2015; Ventura and Todorova, 2015) or threshold crossing counts are sufficient or even better (Stark and Abeles, 2007; Ventura, 2008; Fraser et al., 2009; Chestek et al., 2011; Christie et al., 2015). Though we believe this is the incorrect question to ask, our data suggest that sorted spike counts (without hash) and threshold crossing counts are generally comparable. Our results differed between monkeys (similar to Christie et al., 2015). Our monkeys had different length electrodes, and different experimenters specified the spike detection thresholds and templates for spike sorting. These facts suggest that whether sorted spike counts or threshold crossing counts are better for motor decoding depends on the specifics of the experimental preparation.

We believe the comparison between sorted spike counts and threshold crossing counts is not the correct way to judge the merit of spike sorting, since the presence of hash is a confounding factor. When the presence or absence of hash as a confounding factor is removed, by comparing sorted spike counts vs. merged spike counts, or by comparing sorted spike counts with hash vs. threshold crossings, the benefit of sorting is clear.

The spike detection thresholds we used in the main analyses were fairly low in absolute value, <3 standard deviations of the noise, compared to typical values in the field (particularly when not spike sorting) and the default values seen in commercial spike acquisition systems. However, our lower absolute threshold values were likely more optimal for extracting motor intention, particularly magnitude of intended hand velocity (Oby et al., 2016). Indeed, our follow-up analyses confirm that our original threshold values resulted in better reconstructions than thresholds of −4.5 times noise standard deviation. At the more stringent −4.5 noise standard deviation threshold, sorted spikes with hash was still significantly better than threshold crossings, but sorted was not significantly different from merged.

The noise characteristics of recording setups may differ, so our results here may not generalize. For example, a recording setup which picks up many threshold crossing artifacts may not have a net benefit from including hash. However, our findings suggest that BMI practitioners who use sorted spike counts should examine whether including hash waveforms as a multiunit improves decoding in their experimental preparation.

### Related work

Besides the work of Ventura and Todorova (2015), on which this work is based, there has been other work using waveform features for decoding. Chen et al. (2012) and Kloosterman et al. (2014) used spike waveform features to decode from hippocampal recordings. Their approach is based on the spatial-temporal Poisson process, where a Poisson process describes the spike arrival time and a random vector describes the waveform features of the spike. Later, Deng et al. (2015) presented a marked point-process decoder that uses waveform features as the marks of the point-process and tested it on hippocampal recordings. This approach is similar in spirit to the spatial-temporal Poisson process. The primary difference between these approaches and ours is in the way time is segmented. The spatial-temporal Poisson process and marked point-process operate on single spikes, which requires a high refresh rate and somewhat more sophisticated Bayesian inference. Our approach works on time bins, which allow lower refresh rates and compatibility with the relatively simple Kalman and Wiener filters. However, operating on time bins requires some way to summarize the waveform shape information of all the spikes which occurred during the bin, hence Ventura and Todorova's moments and our sums. These statistics entail their own assumptions (linearity of tuning, stationarity of waveform shape, etc.) and approximations (using a finite number of moments or sums).

Todorova et al. (2014) decoded motor intent from threshold crossing counts and the spike amplitude waveform feature. Their model was non-parametric and fitted using the expectation-maximization scheme of Ventura (2009). Due to the non-parametric model, decoding required a computationally-expensive particle filter. This drawback led to the search for a more computationally efficient method, the result of which is the waveform feature moments framework of Ventura and Todorova (2015).

Also related are earlier work by Ventura on spike sorting using motor tuning information (Ventura, 2009) and on sorting entire spike trains to take advantage of history information (Ventura and Gerkin, 2012). These ideas are similar to waveform feature decoding in that they also combine spike shape information and neural tuning, but different in that the goal is spike sorting.

### Limitations

One limitation of this study is the use of offline reconstructions. There is some debate in the field as to whether improved offline reconstructions always translate into improved online control. Further work using online decoding comparisons are needed to fully verify the efficacy of waveform feature decoding.

## Author contributions

JL and ZL designed the study and wrote the manuscript. JL collected and analyzed the data.

### Conflict of interest statement

The authors declare that the research was conducted in the absence of any commercial or financial relationships that could be construed as a potential conflict of interest.

## References

[B1] Bensmaia S. J. Miller L. E. (2014). Restoring sensorimotor function through intracortical interfaces: progress ands looming challenges. Nat. Rev. Neurosci. 15, 313–325. 10.1038/nrn3724 24739786 PMC12278825

[B2] Carmena J. M. Lebedev M. A. Crist R. E. O'Doherty J. E. Santucci D. M. Dimitrov D. F.. (2003). Learning to control a brain-machine interface for reaching and grasping by primates. PLoS Biol. 1:e42. 10.1371/journal.pbio.0000042 14624244 PMC261882

[B3] Chen Z. Kloosterman F. Layton S. Wilson M. A. (2012). Transductive neural decoding for unsorted neuronal spikes of rat hippocampus, in 2012 Annual International Conference of the IEEE Engineering in Medicine and Biology Society (San Diego, CA), 1310–1313. 10.1109/EMBC.2012.6346178 PMC3972894 23366139

[B4] Chestek C. A. Gilja V. Nuyujukian P. Foster J. D. Fan J. M. Kaufman M. T.. (2011). Long-term stability of neural prosthetic control signals from silicon cortical arrays in rhesus macaque motor cortex. J. Neural Eng. 8:045005. 10.1088/1741-2560/8/4/045005 21775782 PMC3644617

[B5] Christie B. P. Tat D. M. Irwin Z. T. Gilja V. Nuyujukian P. Foster J. D.. (2015). Comparison of spike sorting and thresholding of voltage waveforms for intracortical brain–machine interface performance. J. Neural Eng. 12:016009. 10.1088/1741-2560/12/1/016009 25504690 PMC4332592

[B6] Cunningham J. P. Gilja V. Ryu S. I. Shenoy K. V. (2009). Methods for estimating neural firing rates, and their application to brain–machine interfaces. Neural Netw. 22, 235–1246. 10.1016/j.neunet.2009.02.004 19349143 PMC2783748

[B7] Deng X. Liu D. F. Kay K. Frank L. M. Eden U. T. (2015). Clusterless decoding of position from multiunit activity using a marked point process filter. Neural Comput. 27, 1–23. 10.1162/NECO_a_00744 25973549 PMC4805376

[B8] Fraser G. W. Chase S. M. Whitford A. Schwartz A. B. (2009). Control of a brain-computer interface without spike sorting. J. Neural Eng. 6:055004. 10.1088/1741-2560/6/5/055004 19721186

[B9] Gibson S. Judy J. W. Markovic D. (2012). Spike sorting. IEEE Signal Process. Magaz. 124–143. 10.1109/MSP.2011.941880

[B10] Kloosterman F. Layton S. P. Chen Z. Wilson M. A. (2014). Bayesian decoding using unsorted spikes in the rat hippocampus. J. Neurophysiol. 111, 217–227. 10.1152/jn.01046.2012 24089403 PMC3921373

[B11] Lewicki M. S. (1998). A review of methods for spike sorting: the detection and classification of neural action potentials. Netw. Comput. Neural Syst. 9, 53–78. 10.1088/0954-898X_9_4_001 10221571

[B12] Li S. Li J. Li Z. (2016). An improved unscented kalman filter based decoder for cortical brain-machine interfaces. Front. Neurosci. 10:587. 10.3389/fnins.2016.00587 28066170 PMC5177654

[B13] Li Z. O'Doherty J. E. Lebedev M. A. Nicolelis M. A. L. (2011). Adaptive decoding for brain-machine interfaces through Bayesian parameter updates. Neural Comput. 23, 3162–3204. 10.1162/NECO_a_00207 21919788 PMC3335277

[B14] Nicolelis M. A. (2003). Brain-machine interfaces to restore motor function and probe neural circuits. Nat. Rev. Neurosci. 4, 417–422. 10.1038/nrn1105 12728268

[B15] Oby E. R. Perel S. Sadtler P. T. Ruff D. A. Mischel J. L. Montez D. F.. (2016). Extracellular voltage threshold settings can be tuned for optimal encoding of movement and stimulus parameters. J. Neural Eng. 13:036009. 10.1088/1741-2560/13/3/036009 27097901 PMC5931220

[B16] Perel S. Sadtler P. T. Oby E. R. Ryu S. I. Tyler-Kabara E. C. Batista A. P.. (2015). Single-unit activity, threshold crossings, and local field potentials in motor cortex differentially encode reach kinematics. J. Neurophysiol. 114, 1500–1512. 10.1152/jn.00293.2014 26133797 PMC4556850

[B17] Smith C. Paninski L. (2013). Computing loss of efficiency in optimal bayesian decoders given noisy or incomplete spike trains. Netw. Comput. Neural Syst. 24, 75–98. 10.3109/0954898X.2013.789568 23742213

[B18] Stark E. Abeles M. (2007). Predicting movement from multiunit activity. J. Neurosci. 27, 8387–8394. 10.1523/JNEUROSCI.1321-07.2007 17670985 PMC6673077

[B19] Todorova S. Sadtler P. Batista A. Chase S. Ventura V. (2014). To sort or not to sort: the impact of spikesorting on neural decoding performance. J. Neural Eng. 11:056005. 10.1088/1741-2560/11/5/056005 25082508 PMC4454741

[B20] Ventura V. (2008). Spike train decoding without spike sorting. Neural Comput. 20, 923–963. 10.1162/neco.2008.02-07-478 18085990 PMC3124143

[B21] Ventura V. (2009). Automatic spike sorting using tuning information. Neural Comput. 21, 2466–2501. 10.1162/neco.2009.12-07-669 19548802 PMC4167425

[B22] Ventura V. Gerkin R. (2012). Accurately estimating neuronal correlation requires a new spike-sorting paradigm. Proc. Natl. Acad. Sci. U.S.A. 109, 7230–7235. 10.1073/pnas.1115236109 22529350 PMC3358902

[B23] Ventura V. Todorova S. (2015). A computationally efficient method for incorporating spike waveform information into decoding algorithms. Neural Comput. 27, 1–18. 10.1162/NECO_a_00731 25774541 PMC4884017

[B24] Wheeler B. C. Heetderks W. J. (1982). A comparison of techniques for classification of multiple neural signals. IEEE Trans. Biomed. Eng. BME-29, 752–759. 10.1109/TBME.1982.324870 7173942

[B25] Won D. S. (2007). An Information-Theoretic Analysis of Spike Processing in a Neuroprosthetic Model. Dissertation, Duke University.

[B26] Wu W. Gao Y. Bienenstock E. Donoghue J. P. Black M. (2006). Bayesian population decoding of motor cortical activity using a Kalman filter. Neural Comput. 18, 80–118. 10.1162/089976606774841585 16354382

