# Supplementary material for: Sums of Spike Waveform Features for Motor Decoding
Source: Front Neurosci. 2017 Jul 18;11:406. doi: 10.3389/fnins.2017.00406 (PMC5513987; doi:10.3389/fnins.2017.00406)
Supplement: Supplementary file 1 [file Table1.docx]

Supplementary Material

Sums of Spike Waveform Features for Motor Decoding

Jie Li, Zheng Li*

*** Correspondence:** Zheng Li: lz@bnu.edu.cn

# Supplementary Tables

**Common formatting for all tables: Each column is a comparison of two methods shown in the header. h: signs indicate which value was greater (upper or lower) when significant, 0 indicates not significant. p: p-value of sign test. cp: corrected p-value by the Holm-Bonferroni method (when appropriate). Name code for rows: first letter: monkey {B,M}; second letter: kinematic variable {position, velocity}, third letter: filter {Kalman, Wiener}; fourth letter: metric {correlation, signal-to-noise ratio, mean-squared-error}. Amounts of data were n=47 for monkey B, n=28 for monkey M. F1: amplitude. F2: peak-to-trough time. F3: trough. F4: peak.**

**Supplementary Table A: Significance testing results for amplitude sum.**

| Sign test | | F1_sum | F1_sum | F1_sum | F1_sum | F1_sum |
| --- | --- | --- | --- | --- | --- | --- |
|  |  | Sorted+hash | F123_sum+TC | TC | Sorted | F1_Sum+TC |
| BPKC | h | ∨ | ∨ | ∨ | ∨ | 0 |
|  | p | 0.0400 | 0.0000 | 0.0000 | 0.0000 | 0.1439 |
| BPKS | h | ∨ | ∨ | ∨ | ∨ | 0 |
|  | p | 0.0186 | 0.0000 | 0.0000 | 0.0000 | 1.0000 |
| BVKC | h | 0 | ∨ | ∨ | ∨ | ∨ |
|  | p | 0.2430 | 0.0000 | 0.0000 | 0.0000 | 0.0000 |
| BVKS | h | 0 | ∨ | ∨ | ∨ | ∨ |
|  | p | 0.0789 | 0.0000 | 0.0000 | 0.0000 | 0.0003 |
| BPWC | h | 0 | ∨ | ∨ | ∨ | ∧ |
|  | p | 0.7709 | 0.0186 | 0.0000 | 0.0000 | 0.0001 |
| BPWS | h | 0 | 0 | ∨ | ∨ | ∧ |
|  | p | 0.1439 | 0.3817 | 0.0000 | 0.0000 | 0.0001 |
| BVWC | h | 0 | 0 | ∨ | ∨ | ∧ |
|  | p | 0.7709 | 0.0789 | 0.0000 | 0.0000 | 0.0000 |
| BVWS | h | 0 | 0 | ∨ | ∨ | ∧ |
|  | p | 1.0000 | 0.2430 | 0.0000 | 0.0000 | 0.0000 |
| MPKC | h | 0 | 0 | ∨ | ∨ | 0 |
|  | p | 0.1849 | 0.1849 | 0.0000 | 0.0000 | 0.5716 |
| MPKS | h | 0 | 0 | ∨ | ∨ | ∨ |
|  | p | 0.5716 | 0.8506 | 0.0000 | 0.0000 | 0.0125 |
| MVKC | h | 0 | ∨ | ∨ | ∨ | 0 |
|  | p | 1.0000 | 0.0125 | 0.0002 | 0.0000 | 0.5716 |
| MVKS | h | 0 | 0 | ∨ | ∨ | 0 |
|  | p | 1.0000 | 0.0872 | 0.0002 | 0.0000 | 0.8506 |
| MPWC | h | ∨ | 0 | ∨ | ∨ | ∧ |
|  | p | 0.0357 | 1.0000 | 0.0000 | 0.0000 | 0.0009 |
| MPWS | h | 0 | 0 | ∨ | ∨ | ∧ |
|  | p | 0.0872 | 0.8506 | 0.0000 | 0.0000 | 0.0037 |
| MVWC | h | ∨ | 0 | ∨ | ∨ | ∧ |
|  | p | 0.0002 | 0.1849 | 0.0000 | 0.0000 | 0.0037 |
| MVWS | h | ∨ | 0 | ∨ | ∨ | ∧ |
|  | p | 0.0000 | 0.0872 | 0.0000 | 0.0000 | 0.0357 |

**Supplementary Table B: Significance testing results for moment versus sum comparison.**

| Sign test | | F123_sum+TC | F123_sum | F123_sum |
| --- | --- | --- | --- | --- |
|  |  | F123_moment+TC | F123_moment | F123_moment+TC |
| BPKC | h | ∨ | ∨ | ∨ |
|  | p | 0.0000 | 0.0000 | 0.0000 |
| BPKS | h | ∨ | ∨ | ∨ |
|  | p | 0.0000 | 0.0000 | 0.0003 |
| BVKC | h | ∨ | ∨ | ∨ |
|  | p | 0.0000 | 0.0000 | 0.0000 |
| BVKS | h | ∨ | ∨ | ∨ |
|  | p | 0.0000 | 0.0000 | 0.0000 |
| BPWC | h | ∨ | ∨ | 0 |
|  | p | 0.0031 | 0.0000 | 0.7709 |
| BPWS | h | ∨ | ∨ | 0 |
|  | p | 0.0186 | 0.0000 | 0.7709 |
| BVWC | h | ∨ | ∨ | ∨ |
|  | p | 0.0000 | 0.0000 | 0.0000 |
| BVWS | h | ∨ | ∨ | ∨ |
|  | p | 0.0000 | 0.0000 | 0.0000 |
| MPKC | h | ∨ | ∨ | ∨ |
|  | p | 0.0000 | 0.0000 | 0.0002 |
| MPKS | h | ∨ | ∨ | ∨ |
|  | p | 0.0000 | 0.0000 | 0.0009 |
| MVKC | h | ∨ | ∨ | ∨ |
|  | p | 0.0000 | 0.0000 | 0.0000 |
| MVKS | h | ∨ | ∨ | ∨ |
|  | p | 0.0000 | 0.0000 | 0.0002 |
| MPWC | h | ∨ | ∨ | ∨ |
|  | p | 0.0037 | 0.0000 | 0.0037 |
| MPWS | h | ∨ | ∨ | ∨ |
|  | p | 0.0125 | 0.0000 | 0.0357 |
| MVWC | h | ∨ | ∨ | ∨ |
|  | p | 0.0000 | 0.0000 | 0.0000 |
| MVWS | h | ∨ | ∨ | ∨ |
|  | p | 0.0000 | 0.0000 | 0.0000 |

**Supplementary Table C: Significance testing results for comparison to previous work. Note that higher MSE is worse while higher CC is better.**

| Sign test | | F1_moment  +TC | F1_moment  +TC | F1_moment  +TC | F1_moment  +TC | F1_sum | F1_sum | F1_sum | F1_moment  +TC | F1_sum |
| --- | --- | --- | --- | --- | --- | --- | --- | --- | --- | --- |
|  |  | Sorted | TC | Sorted +hash | F1_sum | Sorted | TC | Sorted  +hash | F1_sum  +TC | F1_sum  +TC |
| BVKM | h | ∧ | ∧ | 0 | ∨ | ∧ | ∧ | ∧ | ∧ | ∧ |
|  | p | 0.0000 | 0.0000 | 0.0789 | 0.0000 | 0.0000 | 0.0000 | 0.0003 | 0.0003 | 0.0003 |
| MVKM | h | ∧ | ∧ | 0 | ∨ | ∧ | ∧ | 0 | 0 | 0 |
|  | p | 0.0000 | 0.0002 | 0.1849 | 0.0125 | 0.0000 | 0.0000 | 0.3449 | 0.8506 | 0.5716 |
| BVKC | h | ∨ | ∨ | ∧ | ∧ | ∨ | ∨ | 0 | ∧ | ∨ |
|  | p | 0.0011 | 0.0000 | 0.0186 | 0.0000 | 0.0000 | 0.0000 | 0.2430 | 0.0000 | 0.0000 |
| MVKC | h | ∨ | ∨ | ∧ | ∧ | ∨ | ∨ | 0 | 0 | 0 |
|  | p | 0.0000 | 0.0125 | 0.0125 | 0.0000 | 0.0000 | 0.0002 | 1.0000 | 0.3449 | 0.5716 |

**Supplementary Table D: Significance testing results for waveform feature comparison. Holm-Bonferroni correction for multiple comparisons was used. cp: corrected p-value by the Holm-Bonferroni method.**

| Sign test | | Amplitude | Amplitude | Amplitude | Peak-to-trough  time | Peak-to-trough  time | Trough |
| --- | --- | --- | --- | --- | --- | --- | --- |
|  |  | Peak-to-trough  time | Trough | Peak | Trough | Peak | Peak |
| BPKC | h | ∨ | ∨ | ∨ | ∧ | ∧ | 0 |
|  | p | 0.0000 | 0.0000 | 0.0000 | 0.0000 | 0.0000 | 0.3817 |
|  | cp | 0.0000 | 0.0000 | 0.0000 | 0.0000 | 0.0000 | 0.3817 |
| BVKC | h | ∨ | ∨ | ∨ | ∧ | ∧ | 0 |
|  | p | 0.0000 | 0.0000 | 0.0000 | 0.0000 | 0.0000 | 0.2430 |
|  | cp | 0.0000 | 0.0000 | 0.0000 | 0.0000 | 0.0000 | 0.2430 |
| MPKC | h | ∨ | ∨ | ∨ | ∨ | ∧ | 0 |
|  | p | 0.0000 | 0.0000 | 0.0002 | 0.0125 | 0.0009 | 0.1849 |
|  | cp | 0.0000 | 0.0000 | 0.0007 | 0.0251 | 0.0027 | 0.1849 |
| MVKC | h | ∨ | ∨ | ∨ | ∨ | ∧ | 0 |
|  | p | 0.0000 | 0.0000 | 0.0009 | 0.0037 | 0.0000 | 0.8506 |
|  | cp | 0.0000 | 0.0000 | 0.0027 | 0.0074 | 0.0001 | 0.8506 |

**Supplementary Table E: Significance testing results for order comparison. Order 2 includes features exponentiated to 1 and 2 before summation, etc. Holm-Bonferroni correction for multiple comparisons was used. cp: corrected p-value by the Holm-Bonferroni method.**

| Sign test | | Order 1 | Order 1 | Order 1 | Order 2 | Order 2 | Order 3 |
| --- | --- | --- | --- | --- | --- | --- | --- |
|  |  | Order 2 | Order 3 | Order 4 | Order 3 | Order 4 | Order 4 |
| BPKC | h | ∧ | 0 | ∧ | ∨ | ∧ | ∧ |
|  | p | 0.0000 | 0.5601 | 0.0000 | 0.0000 | 0.0011 | 0.0000 |
|  | cp | 0.0001 | 0.5601 | 0.0000 | 0.0000 | 0.0022 | 0.0000 |
| BVKC | h | 0 | ∨ | ∧ | ∨ | ∧ | ∧ |
|  | p | 0.3817 | 0.0000 | 0.0003 | 0.0000 | 0.0000 | 0.0000 |
|  | cp | 0.3817 | 0.0000 | 0.0007 | 0.0000 | 0.0000 | 0.0000 |
| MPKC | h | 0 | 0 | 0 | ∨ | 0 | ∧ |
|  | p | 0.3449 | 0.0125 | 1.0000 | 0.0009 | 0.5716 | 0.0009 |
|  | cp | 1.0000 | 0.0502 | 1.0000 | 0.0055 | 1.0000 | 0.0055 |
| MVKC | h | ∨ | ∨ | 0 | ∨ | 0 | ∧ |
|  | p | 0.0125 | 0.0000 | 0.0357 | 0.0002 | 0.8506 | 0.0002 |
|  | cp | 0.0376 | 0.0000 | 0.0714 | 0.0009 | 0.8506 | 0.0009 |

**Supplementary Table F: Significance testing results for raw versus central moment comparison.**

| Sign test | | Raw | Raw+TC |
| --- | --- | --- | --- |
|  |  | Central | Central+TC |
| BPKC | h | ∨ | ∨ |
|  | p | 0.0000 | 0.0000 |
| BVKC | h | ∨ | ∨ |
|  | p | 0.0000 | 0.0000 |
| MPKC | h | ∨ | ∨ |
|  | p | 0.0000 | 0.0000 |
| MVKC | h | ∨ | ∨ |
|  | p | 0.0000 | 0.0000 |

**Supplementary Table G: Significance testing results for sorting versus unsorted comparison.**

| Sign test | | Sorted+hash | Sorted | Sorted | Sorted | Merged |
| --- | --- | --- | --- | --- | --- | --- |
|  |  | TC | Merged | TC | Sorted+hash | TC |
| BPKC | h | ∨ | ∨ | 0 | ∧ | ∧ |
|  | p | 0.0000 | 0.0000 | 1.0000 | 0.0000 | 0.0000 |
| BPKS | h | ∨ | ∨ | 0 | ∧ | ∧ |
|  | p | 0.0000 | 0.0000 | 0.7709 | 0.0000 | 0.0000 |
| BVKC | h | ∨ | ∨ | 0 | ∧ | ∧ |
|  | p | 0.0000 | 0.0000 | 0.3817 | 0.0000 | 0.0000 |
| BVKS | h | ∨ | ∨ | 0 | ∧ | ∧ |
|  | p | 0.0000 | 0.0000 | 0.3817 | 0.0000 | 0.0000 |
| BPWC | h | ∨ | ∨ | 0 | ∧ | ∧ |
|  | p | 0.0000 | 0.0000 | 0.3817 | 0.0000 | 0.0000 |
| BPWS | h | ∨ | ∨ | 0 | ∧ | ∧ |
|  | p | 0.0000 | 0.0000 | 0.7709 | 0.0000 | 0.0000 |
| BVWC | h | ∨ | ∨ | 0 | ∧ | ∧ |
|  | p | 0.0000 | 0.0000 | 0.7709 | 0.0000 | 0.0000 |
| BVWS | h | ∨ | ∨ | 0 | ∧ | ∧ |
|  | p | 0.0000 | 0.0000 | 0.5601 | 0.0000 | 0.0000 |
| MPKC | h | ∨ | ∨ | ∧ | ∧ | ∧ |
|  | p | 0.0000 | 0.0000 | 0.0000 | 0.0000 | 0.0000 |
| MPKS | h | ∨ | ∨ | ∧ | ∧ | ∧ |
|  | p | 0.0000 | 0.0000 | 0.0000 | 0.0000 | 0.0000 |
| MVKC | h | ∨ | ∨ | ∧ | ∧ | ∧ |
|  | p | 0.0000 | 0.0000 | 0.0000 | 0.0000 | 0.0000 |
| MVKS | h | ∨ | ∨ | ∧ | ∧ | ∧ |
|  | p | 0.0000 | 0.0000 | 0.0000 | 0.0000 | 0.0000 |
| MPWC | h | ∨ | ∨ | ∧ | ∧ | ∧ |
|  | p | 0.0002 | 0.0000 | 0.0000 | 0.0000 | 0.0000 |
| MPWS | h | ∨ | ∨ | ∧ | ∧ | ∧ |
|  | p | 0.0000 | 0.0000 | 0.0000 | 0.0000 | 0.0000 |
| MVWC | h | ∨ | ∨ | ∧ | ∧ | ∧ |
|  | p | 0.0000 | 0.0000 | 0.0000 | 0.0000 | 0.0000 |
| MVWS | h | ∨ | ∨ | ∧ | ∧ | ∧ |
|  | p | 0.0002 | 0.0000 | 0.0000 | 0.0000 | 0.0000 |

**Supplementary Table H: Significance testing results comparing methods when using more stringent spike detection thresholds. Methods used -4.5 times noise standard deviation as the spike detection threshold for all channels, unless marked with (original threshold). cp: corrected p-value by the Holm-Bonferroni method.**

| Sign test | | Sorted | Merged | Sorted | Sorted+hash | Sorted | F1_sum | F1_sum | F1_moment  +TC |
| --- | --- | --- | --- | --- | --- | --- | --- | --- | --- |
|  |  | Sorted+hash | TC | Merged | TC | TC | Sorted | TC | F1_sum |
| BVKC | h | ∧ | ∧ | 0 | ∨ | ∧ | ∨ | ∧ | ∧ |
|  | p | 0.0000 | 0.0000 | 0.3817 | 0.0000 | 0.0000 | 0.0000 | 0.0031 | 0.0000 |
| MVKC | h | ∧ | ∧ | 0 | ∨ | ∧ | ∨ | 0 | ∧ |
|  | p | 0.0000 | 0.0000 | 0.5716 | 0.0000 | 0.0000 | 0.0125 | 0.3449 | 0.0000 |
| Sign test | | F1 | F1 | F1 | F2 | F2 | F3 |  | |
|  |  | F2 | F3 | F4 | F3 | F4 | F4 |  |  |
| BVKC | h | ∨ | ∨ | ∨ | ∧ | ∧ | 0 |  |  |
|  | p | 0.0000 | 0.0011 | 0.0003 | 0.0001 | 0.0000 | 0.3817 |  |  |
|  | cp | 0.0000 | 0.0022 | 0.0010 | 0.0004 | 0.0001 | 0.3817 |  |  |
| MVKC | h | ∨ | 0 | 0 | 0 | 0 | 0 |  |  |
|  | p | 0.0000 | 0.0357 | 0.0125 | 0.0357 | 0.0125 | 0.5716 |  |  |
|  | cp | 0.0000 | 0.1071 | 0.0627 | 0.1071 | 0.0627 | 0.5716 |  |  |
| Sign test | | F1_sum (original threshold) | F1_sum  (original threshold) | F1_sum  (original threshold) |  | | | | |
|  |  | Sorted | Sorted  +hash | TC |  |  |  |  |  |
| BVKC | h | ∨ | ∨ | ∨ |  |  |  |  |  |
|  | p | 0.0000 | 0.0000 | 0.0000 |  |  |  |  |  |
| MVKC | h | ∨ | ∨ | ∨ |  |  |  |  |  |
|  | p | 0.0000 | 0.0000 | 0.0000 |  |  |  |  |  |
| Sign test | | Sorted (original threshold) | Sorted+hash  (original threshold) | Merged  (original threshold) | TC  (original threshold) | F1_sum (original threshold) | F2_sum (original threshold) | F3_sum (original threshold) | F4_sum (original threshold) |
|  |  | Sorted | Sorted  +hash | Merged | TC | F1_sum | F2_sum | F3_sum | F4_sum |
| BVKC | h | ∨ | ∨ | ∨ | ∨ | ∨ | ∨ | ∨ | ∨ |
|  | p | 0.0000 | 0.0000 | 0.0000 | 0.0000 | 0.0000 | 0.0000 | 0.0000 | 0.0000 |
| MVKC | h | ∨ | ∨ | ∨ | ∨ | ∨ | ∨ | ∨ | ∨ |
|  | p | 0.0000 | 0.0000 | 0.0000 | 0.0000 | 0.0000 | 0.0000 | 0.0000 | 0.0000 |
